# Effects of maternal BMI on early pregnancy endocrine–metabolic function and offspring development: Evidence from a retrospective cohort and animal model

**DOI:** 10.1371/journal.pone.0333081

**Published:** 2026-01-08

**Authors:** Wendi Shen, Ana Niu, Qiying Zhang, Jialing Deng, Xinya Xu, Xian Ma, Hongli Zhao, Yiyun Lou

**Affiliations:** 1 Department of Gynaecology, Hangzhou TCM Hospital Affiliated to Zhejiang Chinese Medical University, Hangzhou, Zhejiang Province, China; 2 Department of Rehabilitation, Pujiang County Hospital of Traditional Chinese Medicine, Jinhua, Zhejiang Province, China; 3 Huanghu Town Community Health Service Center, Yuhang District, Hangzhou, Zhejiang Province, China; 4 Zhongguan Township Central Hospital, Huzhou, Zhejiang Province, China; 5 Medical School, Zhejiang Chinese Medical University, Hangzhou, Zhejiang Province, China; 6 Key Laboratory of Precise Protection and Promotion of Fertility, Hangzhou, Zhejiang Province, China; Sriwijaya University: Universitas Sriwijaya, INDONESIA

## Abstract

**Objective:**

Abnormal body mass index (BMI) has been associated with pregnancy complications and adverse pregnancy outcomes; however, its clinical significance during early pregnancy remains unclear. This study aimed to investigate the relationships and underlying mechanisms between maternal BMI, reproductive endocrine parameters, and pregnancy outcomes, thereby providing a theoretical basis and clinical guidance for the management of pregnancies in women with abnormal BMI.

**Methods:**

A total of 353 pregnant women were enrolled in this study and categorized into four groups according to BMI: underweight (n = 45), normal weight (n = 229), overweight (n = 57), and obese (n = 22). At 6–8 weeks of gestation, pregnancy-related hormones, glucose and lipid metabolism parameters, and uterine artery blood flow indices were collected and analyzed. Pregnancy outcomes and neonatal birth information were also recorded. In addition, an obese mouse model was established to evaluate maternal weight changes during pregnancy and their effects on uterine and embryonic development. The expression of genes and proteins related to en[docrine metabolism, inflammatory regulation, and angiogenesis was assessed using RT-qPCR and Western blot techniques to further explore the potential mechanisms involved.

**Results:**

During early pregnancy, pregnant women with low BMI exhibited significantly lower estradiol levels (*P* < 0.05). In contrast, women with high BMI had significantly reduced levels of human chorionic gonadotropin, estradiol, and progesterone, along with markedly elevated glucose and lipid metabolism parameters (*P* < 0.05). However, BMI had no significant impact on uterine artery blood flow indices, live birth rate, gestational age, or mode of delivery (*P* > 0.05). High BMI was associated with an increased risk of macrosomia in neonates (*P* < 0.05). In the animal experiments, obese pregnant mice exhibited significantly greater gestational weight gain compared to controls (*P* < 0.05), and their offspring showed a predisposition to obesity. RT-qPCR analysis revealed that, relative to the control group, mRNA expression levels of VEGFA, VEGFR-2, CD31, and SIRT1 were significantly decreased in the obese group (*P* < 0.001), whereas mRNA expression of GLUT1, HIF-1α, TNF-α, IL-6, HMGB1, and TLR4 was significantly upregulated (*P* < 0.01). Western blot results demonstrated that compared with controls, the obese group showed significantly lower expression levels of GLUT4 and phosphorylated IRS1 (*P* < 0.0001), while the expression of HIF-1α and TNF-α was significantly increased (*P* < 0.0001). Additionally, there were no significant differences in the protein expression levels of IRS1, SGK1, and NFκB p65 between the two groups (*P* > 0.05).

**Conclusion:**

Abnormal BMI affects hormonal profiles and glucose and lipid metabolism during early pregnancy. Although it does not significantly impact fetal survival rates, it markedly increases the risks of cesarean delivery, preterm birth, and abnormal neonatal birth weight. These alterations not only pose challenges for postpartum maternal and neonatal health management but may also exert adverse effects on the long-term health of the offspring. Maternal obesity may impair pregnancy outcomes by inhibiting insulin signaling, enhancing proinflammatory responses, inducing a hypoxic microenvironment in the decidual tissue, and suppressing angiogenesis, thereby disrupting maternal–fetal interface homeostasis.

## 1. Introduction

In recent years, with lifestyle changes and increasing social pressures, the prevalence of abnormal body mass index (BMI) among women of reproductive age has shown a steadily rising trend worldwide [1,2]. Abnormal BMI may impair female reproductive function by disrupting endocrine balance, inducing lipotoxicity, oxidative stress, and inflammatory responses, interfering with gene expression, and even causing structural alterations in the ovaries, thereby increasing the risk of infertility, miscarriage, and failure of assisted reproductive technologies [3–6]. A substantial body of research has demonstrated a significant association between elevated maternal BMI and adverse perinatal outcomes [7,8]. Previous studies have reported that abnormal BMI is not only an independent risk factor for spontaneous miscarriage but also markedly increases the incidence of pregnancy complications such as gestational diabetes mellitus, hypertension, and preterm birth [9–11].

Moreover, early pregnancy represents a critical period for embryonic implantation and the initial development of organ systems. Beginning in the sixth week of gestation, the embryo starts to differentiate and form essential systems, including the circulatory, nervous, and urinary systems. During this stage, the embryo is particularly sensitive to external factors; immune rejection or abnormalities in the maternal internal environment can lead to embryonic developmental disorders, congenital malformations, or miscarriage, resulting in significant psychological distress for the pregnant woman [12,13]. Our previous studies have found that pregnancy-related hormonal parameters measured at the seventh week of gestation possess good predictive value for pregnancy outcomes [14].

Therefore, this study selected 6–8 weeks of gestation as the observation window and, by integrating clinical data and animal experiments, comprehensively analyzed the impact and potential mechanisms of maternal BMI on early pregnancy hormonal levels, glucose and lipid metabolism, uterine artery blood flow parameters, pregnancy outcomes, and neonatal birth characteristics. The aim is to provide scientific evidence and clinical guidance for pregnancy management in women with abnormal BMI.

## 2. Human study

### 2.1. Data sources and grouping

This study was approved by the Medical Ethics Committee of Hangzhou Hospital of Traditional Chinese Medicine (Ethics Approval Number: 2024KLL219), and the data were accessed for research purposes from December 31, 2024, to January 10, 2025. The authors have access to identifiable information of individual participants during or after data collection, but we have signed a data confidentiality agreement and will anonymize identifiable information when publishing the data.

The study included women aged 20–40 years in early pregnancy who attended Hangzhou Hospital of Traditional Chinese Medicine from June 1, 2022, to June 1, 2023. Women with a history of chronic diseases, reproductive tract anomalies, multiple pregnancies, ectopic pregnancies, or chromosomal abnormalities were excluded. Ultimately, 353 cases with complete clinical records were collected.

Maternal BMI was calculated based on height and weight measured at admission. According to national standards, participants were classified into four groups: underweight (BMI < 18.5 kg/m^2^, n = 45), normal weight (18.5 ≤ BMI < 24 kg/m^2^, n = 229), overweight (24 ≤ BMI < 28 kg/m^2^, n = 57), and obese (BMI ≥ 28 kg/m^2^, n = 22) [15].

### 2.2. Observational indicators

(i) Clinical parameters assessed at 6–8 weeks of gestation: (a) Pregnancy-related hormones: human chorionic gonadotropin (hCG), estradiol (E_2_), and progesterone (P_4_); (b) Glucose and lipid metabolism parameters: triglycerides (TG), total cholesterol (TC), high-density lipoprotein (HDL), and fasting plasma glucose (FPG). According to the criteria of the International Association of Diabetes and Pregnancy Study Groups (IADPSG) and the reference ranges recommended by the hospital, abnormal values were defined as TG ≥ 1.7 mmol/L, TC ≥ 5.2 mmol/L, HDL ≤ 1.0 mmol/L, and FPG ≥ 5.1 mmol/L [16]. (c) Ultrasound parameters of uterine artery blood flow: pulsatility index (PI), resistance index (RI), and the systolic/diastolic ratio (S/D).
(ii) Pregnancy outcomes: Pregnancy outcome, gestational age at delivery, and mode of delivery. Preterm birth was defined as delivery before 37 weeks of gestation (<260 days), and post-term pregnancy as delivery after 42 weeks of gestation (>293 days) [17].
(iii) Neonatal birth characteristics: Neonatal birth weight. Low birth weight was defined as neonatal weight < 2500 g, and macrosomia (high birth weight, HBW) as neonatal weight > 4000 g [17].

### 2.3. Data collection methods

(i) Serum hormone and glucose-lipid metabolism parameters: Peripheral venous blood samples were collected from pregnant women in the morning after overnight fasting. HCG, E_2_, and P_4_ were measured using an automated chemiluminescence immunoassay analyzer (ADVIA Centaur XP). TG, TC, HDL, and FPG values were determined with an automated biochemical analyzer (BECKMAN COULTER AU5842).
(ii) Uterine artery blood flow parameters: A color Doppler ultrasound diagnostic system (Nuewa R9T) was used, with the probe frequency set at 5–9 MHz, to visualize the color Doppler blood flow signals of the bilateral uterine arteries at the uterine isthmus. Measurements of PI, RI, and S/D were performed and recorded by experienced sonographers at the hospital.
(iii) Pregnancy outcomes and neonatal information collection: Data on pregnancy outcomes and neonatal birth characteristics were obtained through the hospital’s electronic medical record system and telephone follow-up.

## 3. Animal study

### 3.1. Animal sources and housing conditions

All experimental protocols strictly complied with the Guide for the Care and Use of Laboratory Animals published by the U.S. National Institutes of Health and were approved by the Ethics Committee of the Animal Experimental Research Center of Zhejiang Chinese Medical University (Ethics Approval No.: IACUC-20240729–21).

The study used 20 specific pathogen-free (SPF) nulliparous female ICR mice (28 days old, body weight 15 ± 2 g) and 10 male ICR mice of the same age and weight, all provided by Zhejiang Chinese Medical University and purchased from Shanghai Slake Laboratory Animal Co., Ltd. (License No.: SCXK Shanghai 2022−0004). All animals were housed in the SPF-grade animal facility of the Animal Experimental Research Center of Zhejiang Chinese Medical University. Environmental conditions were maintained at 22–24 °C, relative humidity of 55%–65%, and a 12-hour light/dark cycle. Animals had free access to food and water.

### 3.2. Obesity model induction

Ten female ICR mice were randomly selected as the normal weight group and co-housed with male mice while being fed a standard diet (provided by the Animal Research Center of Zhejiang Chinese Medical University). The remaining ten female mice were fed a high-fat diet to induce obesity. The high-fat diet was purchased from Wuxi Fanbo Biotechnology Co., Ltd. and composed of the following: 10% lard, 10% egg yolk powder, 2% cholesterol, 0.1% bile salts, 6% casein, 1.2% premix, and 3% maltodextrin, with the remaining proportion being the basal feed. A significant increase in body weight compared to the normal diet group was considered indicative of successful obesity model establishment. After modeling, all mice were fed a standard diet.

From day 21 of modeling (corresponding to day 49 of age), daily vaginal smears were performed at 9:00 a.m. to monitor the estrous cycle. At 56 days of age, female mice in estrus were paired for mating at a ratio of two females to one male at 17:00 on the same day. The presence of a vaginal plug was checked at 8:00 a.m. the following day. Females with a vaginal plug were considered successfully mated, individually housed, and designated as pregnancy day 1 (PD1).

### 3.3. Specimen collection and outcome measures

#### 3.3.1. General indicators.

The general health status of all experimental mice was monitored daily, including coat luster, locomotor activity, behavioral responses, feeding and excretion, and any abnormal secretions. After pregnancy was confirmed, body weight was measured every other day. Mice were also observed for signs of vaginal bleeding or embryo expulsion.

#### 3.3.2. Embryo-related assessments.

From each group, five pregnant mice were randomly selected. Beginning at 21:00 on PD9, food and water were withdrawn. The following morning at 9:00, the mice were anesthetized via intraperitoneal injection of sodium pentobarbital (40 mg/kg), followed by euthanasia through cervical dislocation. The intact uterus, ovaries, and embryos were carefully removed and rinsed with PBS buffer to eliminate adherent tissues. The gross morphology was examined, and the number of implantation sites and resorbed embryos was recorded. Photographic documentation was performed. Portions of the uterine tissue were placed into 2 mL cryovials and stored at −80 °C for subsequent analyses. The remaining tissues were fixed in Eppendorf tubes containing paraformaldehyde for histological examination.

#### 3.3.3. Pregnancy-related assessments.

The remaining five pregnant mice in each group were maintained on a standard diet until spontaneous delivery. The day of parturition was designated as birth day 0 (BD0). Each pup was individually marked and weighed within 12 hours of birth. Thereafter, body weight was measured every three days for 15 consecutive days, with weights recorded to the nearest 0.1 gram.

#### 3.3.4. RNA isolation and RT-qPCR.

Quantitative PCR primers were designed using Primer Premier 6.0 and Beacon Designer 7.8 software and synthesized by Bioengineering (Shanghai) Co., Ltd. The primer sequences are listed in S1 Table. Total RNA was extracted from uterine tissue using the TRIzol® Plus RNA Purification Kit. RNA concentration, purity, and integrity were assessed by UV spectrophotometry (DU-800) and agarose gel electrophoresis. Complementary DNA (cDNA) was synthesized from total RNA using the SuperScript™ III First-Strand Synthesis SuperMix kit. RT-qPCR was performed on a CFX384 Real-Time PCR Detection System (Bio-Rad, USA). Each 20 μL reaction contained 8.0 μL sterile distilled water (SDW), 10.0 μL Power SYBR® Green Master Mix, 0.5 μL each of forward and reverse primers (10 μmol/L), and 1 μL cDNA template. The amplification protocol was as follows: initial denaturation at 95 °C for 1 min, followed by 40 cycles of 95 °C for 15 s and 63 °C for 25 s. All reactions were performed in triplicate. The mRNA expression levels of vascular endothelial growth factor A (VEGFA), vascular endothelial growth factor receptor-2 (VEGFR-2), platelet endothelial cell adhesion molecule-1 (CD31), glucose transporter 1 (GLUT1), hypoxia-inducible factor 1α (HIF-1α), tumor necrosis factor-α (TNF-α), interleukin-6 (IL-6), high-mobility group box 1 (HMGB1), sirtuin 1 (SIRT1), and toll-like receptor 4 (TLR4) were quantified. Relative mRNA expression levels were normalized to β-actin and calculated using the 2^−ΔΔCt^ method.

#### 3.3.5. Protein extraction and western blot quantification.

Total protein was extracted using a lysis buffer containing protease inhibitors (Thermo Pierce, Cat# 78510). Protein concentration was determined with a BCA Protein Assay Kit (Beyotime Biotechnology, Cat# P0010) following the manufacturer’s instructions. Aliquots of 60 μg protein were separated by 8–12% SDS-PAGE under constant voltage conditions (stacking gel: 60 V; resolving gel: 80 V). Proteins were then transferred onto methanol-activated PVDF membranes (Millipore, Cat# IPVH00010) using Tris-Glycine transfer buffer containing 5% methanol at 100 V constant voltage for 2 hours at 4 °C. Following transfer, membranes were blocked with T-TBS containing 5% BSA for 1 hour at room temperature. Subsequently, membranes were incubated overnight at 4 °C with primary antibodies diluted in T-TBS containing 3% BSA (S2 Table). After primary antibody incubation, membranes were incubated with a Goat anti-Mouse IgG (H + L) secondary antibody (Thermo Pierce, Cat# 31160 or 31210) at a 1:5000 dilution in T-TBS supplemented with 3% BSA for 1 hour at room temperature. After T-TBS washes, protein signals were detected using SuperSignal West Dura Extended Duration Substrate (Thermo Pierce, Cat# 34075) with X-ray film exposure. Band intensities were quantified by Image J 1.8.0 (NIH, USA) using β-actin and TATA binding protein (TBP) as loading controls, with relative expression calculated as (target density/internal reference density) ×10ⁿ. Data represent mean ± standard deviation from triplicate experiments.

## 4. Statistical analysis

Statistical analyses were performed using SPSS version 29.0. The distribution of continuous variables was assessed using the *Shapiro–Wilk test.* Data conforming to a normal distribution were expressed as mean ± standard deviation ($\bar{x}\pm s$ ), and differences between two groups were evaluated using the *independent samples t-test*. For comparisons among multiple groups, *one-way ANOVA* was applied when variances were equal. If significant differences were detected, pairwise comparisons were performed using the *Bonferroni* correction, with *adjusted P values* reported.

For data that exhibited a severely skewed distribution, values were expressed as median and interquartile range [*M* (*P*_25_*, P*_75_)], and group comparisons were conducted using the *Kruskal–Wallis test*. If significant differences were found, pairwise comparisons were also performed using the *Bonferroni* method.Categorical variables were presented as proportions, and inter-group differences were analyzed using the *chi-square (X*^2^*) test* or *Fisher’s exact tes*t as appropriate. The *Bonferroni* correction was applied for multiple comparisons. Asterisks were used to indicate statistical significance relative to the normal control group: ^*^P < 0.05, ^**^P < 0.01, ^***^P < 0.001, ^****^P < 0.0001.

## 5. Results

### 5.1. Clinical study findings

#### 5.1.1. Comparison of baseline characteristics among pregnant women.

As shown in S3 Table, there were no statistically significant differences among the groups regarding menstrual cycle length, history of miscarriage, number of adverse pregnancies, or mode of conception (*P* > 0.05), indicating good comparability among the groups. Notably, women in the overweight group were older and had a shorter duration of menstruation, and these differences were statistically significant (*P* < 0.05).

#### 5.1.2. Abnormal BMI may contribute to lower hCG, E_2_, and P_4_ levels during early pregnancy.

As presented in Table 1, compared with the normal-weight group, the overweight group exhibited significantly lower serum hCG levels across all gestational weeks assessed (*P* < 0.05). Additionally, the other groups showed a similar trend of decreased hCG levels, although these differences did not reach statistical significance (*P* > 0.05).

**Table 1:** Pregnancy hormones at 6-8 weeks of gestation [*M* (*P*_25_, *P*_75_)].

Group	Underweight (n = 45)	Normal weight (n = 229)	Overweight (n = 57)	Obesity (n = 22)	*P* value
**hCG (IU/L)**					
6^th^ week	18535.5 (10388.9,34905.4)	25308.9 (18108.2,37118.7)	18976.2 (9036.8,35190.7)^*^	22492.4 (13631.8,37132.6)	0.006
7^th^ week	72022.1 (39238.5,100218.5)	79830.7 (58074.4,1082321.3)	59255.0 (42047.0,98675.1)^*^	71303.5 (35883.8,100290.7)	0.013
8^th^ week	138278.1 (92902.6,186279.6)	142021.4 (113002.8,176623.4)	124530.4 (88801.1,153757.1)^*^	114393.4 (79985.1,166822.7)	0.007
**E**_**2**_ **(pg/mL)**					
6^th^ week	429.0 (278.1,613.3)^*^	543.5 (363.3,732.0)	475.8 (350.8,552.8)	475.9 (378.0,886.4)	0.013
7^th^ week	656.2 (395.5,1037.0)	838.7 (564.9,1133.3)	665.9 (543.9,910.8)	713.0 (520.2,1099.2)	0.026
8^th^ week	885.9 (577.1,1193.4)^***^	1220.3 (834.4,1607.0)	967.6 (740.6,1156.4)^**^	1059.1 (674.2,1500.9)	<0.0001
**P**_**4**_ **(nmol/L)**					
6^th^ week	90.1 (70.6,106.7)	84.0 (66.7,108.0)	72.3 (58.8,99.7)	81.0 (55.1, 114.9)	0.136
7^th^ week	100.1 (85.6, 122.2)	88.8 (72.7, 114.2)	76.9 (56.7,94.1)^**^	82.4 (68.5,97.5)	< 0.001
8^th^ week	105.0 (89.3,124.5)	92.6 (75.7,121.8)	82.6 (68.9,106.1)	89.1 (77.0,106.5)	0.007

E_2_ levels in the underweight group were significantly lower at both 6 and 8 weeks of gestation (*P* < 0.05). And in the overweight groups decreased significantly at 8 weeks of pregnancy (*P* < 0.01). Although the obese group also demonstrated reduced E_2_ levels, the differences compared with the normal-weight group were not statistically significant (*P* > 0.05).

P_4_ levels tended to be lower across the high-BMI groups, with the overweight group showing a significantly reduced P_4_ concentration at 7 weeks of gestation (*P* < 0.01). Interestingly, P_4_ levels in the underweight group were slightly higher, but no statistically significant difference was observed compared to the normal-weight group (*P* > 0.05).

#### 5.1.3. Women with high BMI in early pregnancy are prone to glucose and lipid metabolism disorders.

As shown in Table 2, pregnant women with high BMI exhibited significantly elevated TG and TC levels (*P* < 0.05). Although there was an overall statistical difference in HDL levels, pairwise comparisons between groups did not reveal significant differences (*P* > 0.05). FPG levels and the prevalence of glucose and lipid metabolism disorders were significantly higher in the obese group compared with the normal-weight group (*P* < 0.05), and the incidence of these abnormalities demonstrated a positive correlation with BMI.

**Table 2:** Glycolipid metabolism at 6-8 weeks of gestation [*M* (*P*_25_*, P*_75_)].

Group	Underweight (n = 45)	Normal weight (n = 229)	Overweight (n = 57)	Obesity (n = 22)	*P value*
TG (mmol/L)	0.77 (0.58,0.97)	0.77 (0.57,1.08)	1.02 (0.79,1.28)^**^	1.09 (0.81,1.32)^*^	< 0.0001
TC (mmol/L)	3.90 (3.45,4.35)	3.90 (3.55,4.40)	4.20 (3.70,4.50)	4.25 (3.90,4.73)^*^	0.012
HDL (mmol/L)	1.05 (0.92,1.31)	1.05 (0.92,1.21)	0.98 (0.89,1.12)	1.00 (0.86,1.15)	0.039
Dyslipidemia rate	20 (44.44%)	104 (45.41%)	35 (61.40%)^*^	16 (72.72%)^*^	0.019
FPG (mmol/L)	4.25 (3.91,4.51)	4.32 (4.01,4.65)	4.47 (4.24,4.76)	4.60 (4.33,4.93)^*^	0.002
Abnormal blood glucose rate	1 (2.22%)	7 (3.06%)	5 (8.77%)	5 (22.73%)^***^	< 0.001

#### 5.1.4. Impaired uterine microcirculation in women with high BMI at 6–8 weeks of gestation.

As shown in Table 3, during gestational weeks 6–8, the uterine artery blood resistance flow exhibited a positive correlation with BMI classification. The PI showed a significant difference in overall distribution (*P* < 0.05); however, pairwise comparisons between groups did not reveal statistically significant differences (*P* > 0.05).

**Table 3:** Uterine arterial blood flow at 6-8 weeks of gestation [*M* (*P*_25_*, P*_75_)].

Group	Underweight (n = 45)	Normal weight (n = 229)	Overweight (n = 57)	Obesity (n = 22)	*P value*
RI	0.85 (0.82,0.87)	0.86 (0.82,0.88)	0.87 (0.83,0.90)	0.87 (0.83,0.89)	0.092
PI	2.28 (2.04,2.56)	2.39 (2.10, 2.63)	2.55 (2.14,2.83)	2.60 (2.12,2.85)	0.047
S/D	6.56 (5.48,8.28)	7.10 (5.71,8.79)	7.37 (6.01,10.31)	7.63 (5.92,9.57)	0.181
The sum of S/D on both sides	13.12 (10.95,16.55)	14.20 (11.42,17.58)	14.74 (12.02,20.61)	15.25 (11.84,19.14)	0.181

#### 5.1.5. Maternal BMI affects neonatal birth outcomes.

As presented in Table 4, there were no statistically significant differences among groups regarding live birth rate, gestational age at delivery, mode of delivery, incidence of LBW, preterm birth rate, or overall neonatal abnormalities (*P* > 0.05). Additionally, no cases of post-term pregnancy were observed in any group.

**Table 4:** Pregnancy outcomes and neonatal conditions [$\bar{x}\pm s$, *n* (%)].

Group	Underweight (n = 45)	Normal weight (n = 229)	Overweight (n = 57)	Obesity (n = 22)	*P value*
Survival	38 (84.44%)	186 (81.22%)	44 (77.19%)	16 (72.73%)	0.623
Abortion	7 (15.56%)	43 (18.78%)	13 (22.81%)	6 (27.27%)	
Group	Underweight (n = 38)	Normal weight (n = 186)	Overweight (n = 44)	Obesity (n = 16)	*P value*
Gestational age	38.00 ± 1.32	38.16 ± 1.47	38.41 ± 1.19	38.06 ± 1.06	0.579
Cesarean section	15 (39.47%)	90 (48.39%)	24 (54.55%)	11 (68.75%)	0.218
Weight (g)	3065.39 ± 356.97	3130.13 ± 397.42	3333.86 ± 341.86^*^	3418 ± 476.26^*^	< 0.001
LBW	2 (5.26%)	8 (4.30%)	1 (2.27%)	0 (0.00%)	0.899
HBW	0 (0.00%)	2 (1.06%)	2 (4.55%)	2 (12.50%)^*^	0.026
Preterm birth	4 (10.53%)	11 (5.91%)	3 (6.82%)	1 (6.25%)	0.674
Total bad conditions	4 (10.53%)	15 (8.06%)	5 (11.36%)	3 (18.75%)	0.396

However, neonatal birth weight and the incidence of HBW were significantly higher in the high-BMI groups (*P* < 0.05). The cesarean section rate was also higher, although the difference was not statistically significant (*P* > 0.05). Both increased neonatal birth weight and macrosomia were positively correlated with maternal BMI.

### 5.2. Results of animal experiments

#### 5.2.1. Comparison of body weight during model induction.

After 21 days of modeling, the body weight of mice in the obesity group was significantly higher compared to the normal group (*P* < 0.0001) (S1 Fig.a). The overall increase in body weight was also significantly greater (*P* < 0.0001) (S1 Fig.b), confirming the successful establishment of the obesity model (S1 Fig.c).

Following model induction, vaginal exfoliated cells were collected daily at 9:00 a.m. During estrus, a large number of cornified epithelial cells and a small number of nucleated epithelial cells were observed. Comparison of vaginal smears between the two groups showed that the number of cornified epithelial cells was reduced in the obesity group, suggesting a shortened estrous phase(S1 Fig.d).

#### 5.2.2. Obesity does not affect maternal weight gain, embryo weight, or embryo loss rate.

**Fig 1:**
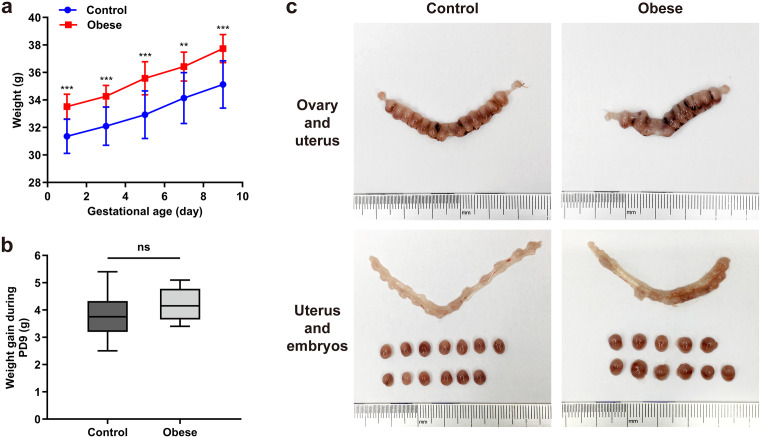
Body weight gain during pregnancy and morphological comparison of uteri and embryos collected on gestational day 10 in mice. **(a)** Line graph depicting maternal body weight at different gestational ages. Group differences are indicated as ^**^P < 0.01, ^***^P < 0.001. **(b)** Box plot showing body weight gain of pregnant mice during PD9. No significant difference was observed between groups (*P* > 0.05). **(c)** Representative images of uteri and implantation sites in pregnant mice (upper panel: uterus, embryos, and ovaries; lower panel: uterus and embryos). Scale bar = 5 mm.

In early pregnancy, the body weight of obese pregnant mice remained significantly higher than that of normal-weight pregnant mice (*P* < 0.01) (Fig 1a); however, the magnitude of weight gain showed no statistically significant difference between groups (*P* > 0.05) (Fig 1b).

There were no significant differences between the obesity and control groups in terms of uterine weight, mean embryo weight, or embryo loss rate (*P* > 0.05) (Table 5). Total uterine weight was associated with the number of implanted embryos. Obese mice had a lower average number of embryos compared to control group.

**Table 5:** Uterine weight and embryo loss rate in mice [$\bar{x}\pm s$, *n*%].

Group	Normal weight (n = 5)	Obese (n = 5)	*P value*
Total uterine weight (g)	0.874 ± 0.085	0.841 ± 0.102	0.594
Net uterine weight (g)	0.267 ± 0.023	0.278 ± 0.016	0.423
Average embryo weight (g)	0.048 ± 0.003	0.051 ± 0.003	0.231
Normal embryo number	12.60 ± 1.14	11.20 ± 2.05	0.219
Dead embryo number	0.00 ± 0.00	0.40 ± 0.55	0.178
Embryo loss rate (%)	0.00	3.40	0.440

In normal-weight mice, the uteri appeared pale red, bead-like in shape, and relatively uniform in size, with occasional minor congestion in the uterine cavity. In contrast, uteri and embryos from obese mice were darker in color, with more pronounced intrauterine congestion and uneven degrees of embryonic development ((Fig 1c).

#### 5.2.3. Offspring of obese pregnant mice exhibit a tendency toward obesity.

**Fig 2:**
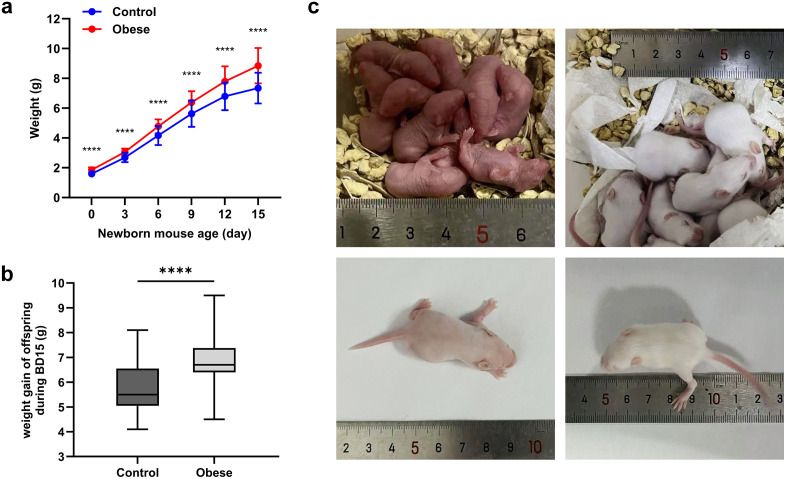
Body weight and morphological changes in neonatal mice. **(a)** Line graph showing body weight changes of neonatal mice at different postnatal ages. Group differences are indicated as ^****^P < 0.0001. **(b)** Box plot depicting total body weight gain during BD15 in neonatal mice. Group differences are indicated as ^****^P < 0.0001. **(c)** Representative morphology of neonatal mice at different postnatal ages. Scale bar = 5 mm.

The body weight of the offspring born to obese pregnant mice was significantly higher than that of the control group (*P* < 0.0001) (Fig 2a). Moreover, during development, these offspring showed a markedly greater increase in body weight, demonstrating an apparent trend toward obesity (*P* < 0.0001) (Fig 2b). The developmental progression of the offspring is illustrated in Fig 2c.

#### 5.2.4. Obesity may induce characteristic endometrial changes of inflammation–hypoxia–metabolic–angiogenic imbalance.

**Fig 3:**
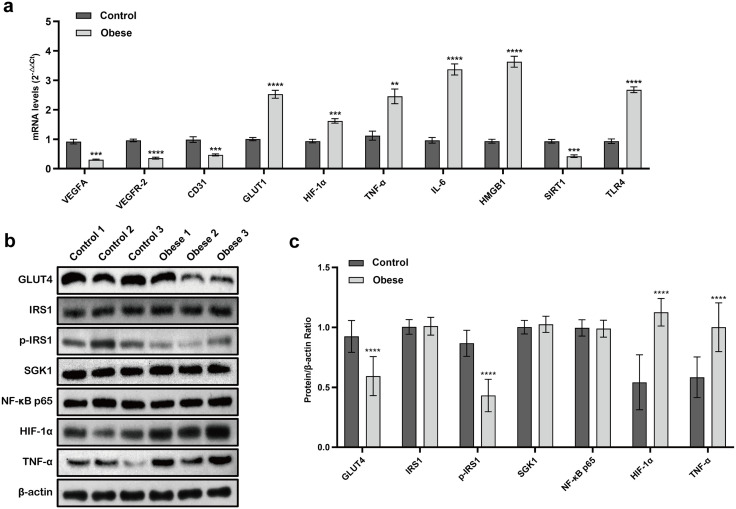
Relative expression levels of different genes and proteins in uterine tissues collected at pregnancy day 10 in mice. **(a)** Relative mRNA expression levels of VEGFA, VEGFR-2, CD31, GLUT1, HIF-1α, TNF-α, IL-6, HMGB1, SIRT1, and TLR4 in uterine tissues analyzed by RT-qPCR (n = 3 per group). Values are presented as mean ± standard deviation. Group differences are indicated as ^**^P < 0.01, ^***^P < 0.001, and ^****^P < 0.0001. **(b)** Representative Western blot bands of GLUT4, IRS1, p-IRS1, SGK1, NF-κB p65, HIF-1α, and TNF-α proteins in uterine tissues (n = 3 per group). **(c)** Densitometric analysis of relative protein expression levels of GLUT4, IRS1, p-IRS1, SGK1, NF-κB p65, HIF-1α, and TNF-α measured by Western blotting (n = 3 per group). Values are presented as mean ± standard deviation. Group differences are indicated as ^****^P < 0.0001.

RT-qPCR results showed that, compared with the control group, mRNA expression levels of VEGFA, VEGFR-2, CD31, and SIRT1 were significantly decreased in the obesity group (*P* < 0.001), while the expression levels of GLUT1, HIF-1α, TNF-α, IL-6, HMGB1, and TLR4 were significantly upregulated (*P* < 0.01) (Fig 3a).

Western blot analysis revealed that, compared with controls, the protein expression levels of GLUT4 and p-IRS1 were markedly decreased in the obesity group (*P* < 0.0001), whereas HIF-1α and TNF-α protein levels were significantly increased (*P* < 0.0001). Additionally, there were no significant differences between groups in the expression of IRS1, SGK1, and NFκB p65 proteins (*P* > 0.05) (Fig 3b and 3c).

## 6. Analysis and discussion

Abnormal BMI is closely associated with various reproductive complications; however, the underlying links between BMI and pregnancy outcomes remain incompletely understood. This study aimed to investigate the relationships between BMI and multiple clinical indicators in early pregnancy, to analyze their potential impact and mechanisms on pregnancy outcomes and fetal development, and to provide a basis for clinical assessment and pregnancy management in women with abnormal BMI.

During pregnancy, placental cholesterol and maternal lipoprotein-derived cholesterol serve as major precursors for the synthesis of sex hormones [18]. Dysregulation of adipose tissue-derived factors can disrupt glucose and lipid metabolism, trigger chronic low-grade inflammation, and alter the immunological microenvironment at the maternal–fetal interface [19]. Concurrently, disruption of metabolic homeostasis impairs adipocyte function and tissue remodeling capacity, further exacerbating maternal inflammatory responses and oxidative stress [20]. These pathological changes can interfere with the function of the hypothalamic–pituitary–ovarian (HPO) axis, suppress the secretion of gonadotropin-releasing hormone (GnRH), and reduce the responsiveness of granulosa cells to follicle-stimulating hormone (FSH), ultimately leading to decreased levels of E_2_ and P_4_ [19,21,22]. These hormonal alterations negatively impact endometrial receptivity and embryo implantation [23,24]. Therefore, the metabolic status of glucose and lipids in early pregnancy may serve as an early warning indicator of metabolic syndrome and adverse pregnancy outcomes [25,26].

In early pregnancy, normal embryonic development depends on a variety of hormones secreted by the corpus luteum and placenta. Among them, hCG promotes corpus luteum growth and estrogen secretion by stimulating the expression of adhesion molecules, improves local uterine blood supply, thereby enhancing endometrial receptivity and facilitating successful embryo implantation [27]. During normal pregnancy, serum hCG levels typically rise significantly starting from gestational week 6 and reach a peak around week 10. Previous studies have demonstrated that hCG levels in early pregnancy are closely associated with pregnancy outcomes [28]. E_2_, a steroid hormone, is primarily secreted by the corpus luteum under the stimulation of hCG during early pregnancy. Its level serves as an important indicator for evaluating dominant follicle quality and luteal function. E_2_ plays a critical role in regulating endometrial receptivity, microcirculation at the maternal–fetal interface, and pregnancy-related immune responses [29,30]. Earlier studies have shown that E_2_ can activate serum- and glucocorticoid-inducible kinase 1 (SGK1), an upstream regulator of the PI3K signaling pathway, effectively inhibiting Toll-like receptor 4 (TLR4)-mediated pro-apoptotic and pro-inflammatory Th1-type immune responses while enhancing anti-inflammatory Th2-type immune responses. This process promotes the differentiation of M2 macrophages, facilitating decidual remodeling and angiogenesis at the maternal–fetal interface during early pregnancy, thereby maintaining an optimal environment for embryo implantation [31,32]. In addition, E_2_ has been reported to enhance the transcriptional activity of the low-density lipoprotein receptor (LDLR) gene and to augment the function of placental desmolase, thereby accelerating the conversion of cholesterol to pregnenolone and promoting P_4_ synthesis [33,34]. Further clinical studies have found that starting from gestational week 6, serum E_2_ levels in women experiencing spontaneous miscarriage are significantly lower compared with those with normal pregnancies [35]. A multicenter study involving 548 singleton pregnancies also confirmed a significant negative correlation between BMI and E_2_ levels, indicating that for every one-unit increase in BMI, E_2_ levels decrease on average by 1%–2% [36]. P_4_ also plays a pivotal role in trophoblast adhesion and proliferation. Working synergistically with E_2_, P_4_ participates in regulating uterine microcirculation and enhances immune tolerance at the maternal–fetal interface, thereby maintaining a stable intrauterine environment necessary for embryo implantation and development [37,38]. Insufficient P_4_ secretion can impair trophoblast invasiveness, increase the risk of maternal immune rejection of the embryo, and ultimately compromise pregnancy maintenance [39].

In terms of endocrine metabolism, GLUT4 is a key transporter regulated by insulin that plays a critical role in cellular energy metabolism by facilitating glucose uptake [40]. IRS1, as a principal mediator of insulin signaling, undergoes tyrosine phosphorylation to activate the phosphoinositide 3-kinase (PI3K)/Akt signaling pathway, thereby promoting GLUT4 translocation to the plasma membrane and enhancing cellular glucose uptake capacity [41,42]. The findings of this study demonstrated that, under obese conditions, the expression levels of GLUT4 and p-IRS1 were significantly downregulated. This is consistent with previous reports indicating that obesity-associated lipid accumulation in the endometrium can suppress IRS1 and GLUT4 expression [43]. Animal experiments by Long et al. also revealed that impaired GLUT4 function leads to restricted glucose uptake by the endometrium during early pregnancy, inducing endometrial dysfunction and reducing embryo implantation rates [40]. Meanwhile, local metabolic disturbances can exacerbate hypoxia in endometrial tissues and induce the upregulation of HIF-1α expression. HIF-1α is a central cellular responder under hypoxic conditions and can enhance the biological effects of VEGF through multiple mechanisms to adapt to a low-oxygen environment [44]. VEGF, a critical downstream target of HIF-1α, promotes endothelial cell migration, proliferation, and angiogenesis [45]. Inactivation of the VEGF gene results in severe vascular developmental defects and is a major cause of early embryonic lethality [46].

Regarding inflammation regulation, HMGB1 is an endogenous danger-associated molecular pattern protein that can be actively or passively released upon cellular damage or necrosis, inducing sterile inflammation and contributing to the dynamic regulation of the inflammatory response and tissue repair [47]. Upon binding to the Toll-like receptor 4 (TLR4), HMGB1 activates the nuclear factor-κB (NF-κB) signaling pathway, increasing the production of pro-inflammatory cytokines such as IL-6 and TNF-α, thereby creating a chronic sterile inflammatory microenvironment [48]. This, in turn, restricts nutrient transport across the placenta, induces decidual cell dysfunction, and disrupts maternal–fetal immune tolerance [49,50]. Yeung et al. reported that under physiological conditions, SIRT1 deacetylates the p65 subunit of NF-κB and suppresses its activity, thereby exerting anti-inflammatory effects [51].

The results of this study showed that underweight pregnant women exhibited slightly lower serum hCG levels compared to those with normal weight, which may be related to delayed embryonic development due to insufficient nutritional intake. Moreover, E_2_ levels in underweight pregnant women were significantly lower than in other groups, whereas P_4_ levels were relatively higher, although the latter did not reach statistical significance. Given that low body weight may lead to dysregulation of reproductive endocrine function and adversely affect ovarian activity and luteal hormone secretion [52], we speculate that reduced E_2_ levels during early pregnancy could be associated with potential ovarian insufficiency in underweight women. Conversely, women with high BMI generally exhibited lower serum levels of hCG, E_2_, and P_4_. This may result from the hemodilution effect associated with obesity as well as its negative impact on ovarian and placental function. However, it is noteworthy that during weeks 6–8 of gestation, pregnancy-related hormone levels in obese women were actually higher than those observed in overweight women. This observation contrasts with the findings of Bartha et al., who reported lower progesterone concentrations in obese women between gestational weeks 9–11 [18]. Lassance et al. further demonstrated that maternal obesity during pregnancy can downregulate mitochondrial cholesterol transport protein TSPO, thereby suppressing mitochondrial steroidogenesis and impairing sex hormone synthesis [53]. Nevertheless, whether a threshold difference exists in cholesterol metabolism or steroid production between overweight and obese populations remains unclear and warrants further investigation.

Additionally, our results showed that women with high BMI had significantly elevated levels and rates of abnormal glucose and lipid metabolism during early pregnancy, with a positive correlation observed between BMI and metabolic disturbance. Obesity-related metabolic disorders can substantially affect the activity of the fibrinolytic system, constituting a major risk factor for both arterial and venous thrombosis [54]. During pregnancy, the uterine spiral arteries undergo invasive remodeling mediated by extravillous trophoblasts (EVTs), which, together with the formation of the intervillous space, establish an efficient perfusion network supplying the embryo with essential oxygen and nutrients to meet metabolic demands throughout gestation [55]. Uterine artery blood flow parameters are widely used to assess uteroplacental circulation during early pregnancy and serve as important indicators of placental perfusion status [56]. Perdu et al. proposed that maternal obesity may impair vascular remodeling at the uteroplacental interface by inhibiting natural killer (NK) cell–mediated remodeling of uterine arteries and interfering with normal trophoblast differentiation [57]. Our study further found a positive correlation between maternal BMI and uterine artery blood flow resistance, suggesting that obese pregnant women are more prone to uterine microcirculatory dysfunction. This finding is highly consistent with previous related studies [58]. Therefore, it is recommended that pregnant women with high BMI undergo enhanced assessment and monitoring of uteroplacental microcirculation function early in pregnancy to prevent adverse pregnancy outcomes associated with inadequate uterine perfusion or thrombotic events.

Regarding pregnancy outcomes and neonatal status, our study found that underweight pregnant women were more prone to preterm birth and delivering low birth weight infants. This observation is consistent with conclusions from previous large-scale studies [59]. Such adverse outcomes are likely related to factors including maternal malnutrition, intrauterine growth restriction (IUGR), and cervical insufficiency. In contrast, neonates born to obese mothers typically had higher birth weights, with a significantly increased risk of macrosomia. This, in turn, raises the likelihood of obstetric complications such as difficult labor and intrapartum hemorrhage and represents one of the major contributors to elevated cesarean section rates. Additionally, previous research has demonstrated that maternal obesity can influence offspring body weight and endocrine–metabolic function through genetic and epigenetic mechanisms [21]. Therefore, it is essential to prioritize BMI management during the preconception period by promoting balanced nutrition and appropriate exercise to optimize weight control strategies. For pregnant women with elevated BMI, it is recommended to closely monitor glucose and lipid metabolism and track gestational weight gain. When necessary, dietary interventions emphasizing low glycemic load and reduced fat intake should be implemented to lower the risk of gestational diabetes mellitus, gestational hypertension, and other adverse pregnancy outcomes.

The RT-qPCR and Western blot analyses in this study showed that obesity may significantly increase miscarriage risk by suppressing insulin signaling and angiogenic networks while activating inflammatory and oxidative stress–related pathways, thereby altering the metabolic and immune microenvironment of decidual tissue. Obesity-related systemic low-grade inflammation can upregulate TLR4 expression via fatty acids and endotoxins, which, in turn, promote HMGB1 release (either passively or actively). This process further activates the TLR4/NF-κB signaling cascade, significantly increasing transcription of pro-inflammatory cytokines such as TNF-α and IL-6. Persistently elevated levels of inflammatory mediators not only maintain a chronic inflammatory state but also suppress SIRT1 transcription and activity. The resulting loss of SIRT1-mediated deacetylation of NF-κB amplifies sustained inflammatory activation. Chronic inflammation and oxidative stress stimulate HIF-1α accumulation, reflecting local uterine hypoxic stress. However, unlike the typical adaptive hypoxic response, high-fat diet–induced inflammation and metabolic disturbances impair the functional regulation of angiogenic genes by HIF-1α, leading to downregulation of VEGFA and its receptor VEGFR-1, which suppresses endothelial cell proliferation and neovascularization. The reduced expression of CD31 further indicates compromised remodeling of the uterine vascular network and diminished angiogenic capacity. The observed upregulation of GLUT1 expression, consistent with increased HIF-1α, suggests that glucose transport is enhanced via insulin-independent pathways in response to localized hypoxia. In contrast, the decreased expression of GLUT4 and phosphorylated IRS1 proteins reflects impaired insulin signaling and a shift away from normal insulin-dependent glucose metabolism. While total IRS1 levels remained unchanged, reduced p-IRS1 levels indicate suppressed activation status, highlighting significant insulin resistance within uterine tissue. Notably, SGK1 expression showed no marked difference, suggesting a limited role in mediating insulin resistance in this context.

To our knowledge, this study is the first to systematically analyze the association between early pregnancy clinical indicators and maternal BMI. However, our investigation primarily focused on differences in key indicators across BMI groups, and the underlying mechanistic relationships among these markers warrant further exploration. Additionally, as a retrospective study, there are inherent limitations, such as the inability to obtain more comprehensive metabolic, inflammatory, and immunological data, and the difficulty in fully excluding the effects of concomitant medications or other confounding factors on the results. Therefore, future prospective, multicenter, large-sample mechanistic studies are needed to validate these findings and elucidate their causal relationships.

## 7. Conclusion

The results of this study demonstrated that during gestational weeks 6–8, underweight pregnant women exhibited relatively lower serum levels of hCG and E_2_, while P_4_ levels were comparatively higher. In contrast, women with high BMI showed a general reduction in all three hormonal indicators. Additionally, obese pregnant women presented with elevated uterine artery resistance indices and significant glucose and lipid metabolic disturbances. These findings provide important clinical reference values for interpreting early pregnancy hormonal and metabolic profiles in women with varying BMI categories.

It is noteworthy that the influence of maternal BMI on early embryonic survival appears to be relatively limited, which may help to alleviate concerns among pregnant women with abnormal BMI. However, deviations from a normal BMI remain strongly associated with an elevated risk of adverse pregnancy outcomes in later gestation, including preterm birth, LBW, HBW, and cesarean delivery. Importantly, while obesity may have a genetic basis, epigenetic modifications shaped by lifestyle, nutrition, and environmental exposures can interact with these genetic susceptibilities, potentially influencing endocrine and metabolic health in the offspring.

Moreover, obesity exhibits a certain degree of metabolic and genetic predisposition. Maternal obesity may induce characteristic alterations in endometrial and placental tissues, manifesting as a dysregulated interplay of inflammation, hypoxia, metabolic imbalance, and impaired angiogenesis. Chronic obesity-related inflammation activates the HMGB1/TLR4/NF-κB signaling cascade, while concurrent downregulation of SIRT1 further amplifies pro-inflammatory signaling. Although hypoxic stress results in upregulation of HIF-1α, it fails to effectively induce angiogenesis. Impairment of insulin signaling leads to downregulation of GLUT4 and disrupts glucose utilization. This multidimensional dysregulation not only damages decidual function and adaptive vascular remodeling but may also compromise maternal–fetal immune tolerance and energy homeostasis, thereby constituting an important molecular basis for adverse pregnancy outcomes.

Therefore, it is recommended to strengthen BMI management before and during pregnancy, with close monitoring of glucose–lipid metabolism and inflammatory markers. Rational control of gestational weight gain through nutritional counseling and lifestyle interventions is essential to prevent obesity-related pregnancy complications and improve maternal and neonatal outcomes.

## 8. Limitations

This study has several limitations that warrant consideration and should be addressed in future research. First, due to its retrospective design, the sample size was constrained by the availability of existing clinical data. In an effort to maximize the sample size, potential confounding variables such as dietary patterns, educational level, and occupation were not controlled, which may have introduced selection bias. Second, the relatively small number of obese pregnant participants may have limited the statistical power and reduced the generalizability of the findings. Third, owing to the retrospective nature of the study, there was a lack of systematic monitoring of body weight dynamics and standardized specimen collection during pregnancy. To address this gap, we employed a mouse model for weight assessment and mechanistic exploration. However, due to species-specific differences between mice and humans, the translational relevance of animal findings may be limited. Therefore, interpretations of the animal data should be made with caution and considered only as preliminary references for understanding the potential mechanisms underlying gestational weight management.

## Supporting information

S1 File Data. (XLSX)

S1 Table RT-qPCR Primer Information. (DOCX)

S2 Table Primary Antibody Information. (DOCX)

S3 Table Basic information on pregnant women. (DOCX)

S1 Fig Changes in body weight and vaginal smears during the modeling process in mice. (a) Line graph showing body weight gain of female mice during the modeling period. ^**^P < 0.01, ^****^P < 0.0001. (b) Box plot illustrating body weight increase in the two groups after modeling. ^****^P < 0.0001. (c) Morphological appearance of mice after modeling (left: control group; right: obese group). (d) Vaginal cytology smears obtained during the modeling process. Scale bar = 40 μm.
(TIF)
