# Supplementary material for: Effects of maternal BMI on early pregnancy endocrine–metabolic function and offspring development: Evidence from a retrospective cohort and animal model
Source: PLoS One. 2026 Jan 8;21(1):e0333081. doi: 10.1371/journal.pone.0333081 (PMC12782434; doi:10.1371/journal.pone.0333081)
Supplement: S1 Table — (DOCX) [file pone.0333081.s002.docx]

**S1 Table. RT-qPCR Primer Information**

| Gene | Genbank Accession | Primer Sequences(5'to3') | Size(bp) | Annealing (℃) |
| --- | --- | --- | --- | --- |
| Mouse β-actin | NM_007393.3 | F: GTGTGACGTTGACATCCGTAAAGA | 247 | 60 |
|  |  | R: GCCGGACTCATCGTACTCCT |  |  |
| Mouse VEGFA | NM_009505 | F: GCACATAGGAGAGATGAGCTTCC | 105 | 60 |
|  |  | R: CTCCGCTCTGAACAAGGCT |  |  |
| Mouse VEGFR-2 | NM_010612.3 | F: CCTGCCTACCTCACCTGTT | 114 | 60 |
|  |  | R: CTCTTTCGCTTACTGTTCTGGAGAT |  |  |
| Mouse CD31 | NM_008816.3 | F: CCAAAGCCAGTAGCATCATGGT | 144 | 60 |
|  |  | R: GGATGGTGAAGTTGGCTACAG |  |  |
| Mouse GLUT1 | XM_006502908.2 | F: GTGTATCCTGTTGCCCTTCTG | 151 | 60 |
|  |  | R: CTGCCGACCCTCTTCTTTC |  |  |
| Mouse HIF-1α | AF003695.1 | F: AGATGACGGCGACATGGTTTAC | 156 | 60 |
|  |  | R: CTCACTGGGCCATTTCTGTGT |  |  |
| Mouse TNFα | NM_013693 | F: GACCCTCACACTCAGATCATCTTCT | 63 | 60 |
|  |  | R: GCTACGACGTGGGCTACAG |  |  |
| Mouse IL-6 | NM_031168 | F: GCCTTCTTGGGACTGATGCTGGT | 92 | 60 |
|  |  | R: CTGTTGGGAGTGGTATCCTCTGTGA |  |  |
| Mouse HMGB1 | NM_010439.4 | F: CCAAGAAGTGCTCAGAGAGGTG | 149 | 60 |
|  |  | R: GTCCTTGAACTTCTTTTTGGTCTC |  |  |
| Mouse SIRT1 | NM_019812.3 | F: GGGAACCTTTGCCTCATCTACATT | 90 | 60 |
|  |  | R: CACCACCTAGCCTATGACACA |  |  |
| Mouse TLR4 | NM_021297.2 | F: GGCATGGCATGGCTTACACCA | 134 | 60 |
|  |  | R: GAGAGGCCAATTTTGTCTCCACA |  |  |

Notes: F indicates the forward primer; R indicates the reverse primer.
