# Supplementary material for: Effects of maternal BMI on early pregnancy endocrine–metabolic function and offspring development: Evidence from a retrospective cohort and animal model
Source: PLoS One. 2026 Jan 8;21(1):e0333081. doi: 10.1371/journal.pone.0333081 (PMC12782434; doi:10.1371/journal.pone.0333081)
Supplement: S2 Table — (DOCX) [file pone.0333081.s003.docx]

**S2 Table. Primary Antibody Information**

| Primary Antibody | Manufacturer (Cat. No.) | Dilution | Molecular Weight (kDa) |
| --- | --- | --- | --- |
| β-actin | Abcam ab8226 | 1/2000 | 42 |
| GLUT4 | Proteintech 66846-1-IgG | 1/2000 | 50 |
| IRS1 | CST 3407 | 1/1000 | 180 |
| p-IRS1 | CST 2381 | 1/1000 | 180 |
| SGK1 | Abcam ab32374 | 1/500 | 57 |
| NF-κB p65 | CST 8242 | 1/1000 | 65 |
| HIF-1α | CST 36169 | 1/1000 | 120 |
| TNFα | Abcam ab6671 | 1/1000 | 26 |
