# Supplementary material for: Effects of maternal BMI on early pregnancy endocrine–metabolic function and offspring development: Evidence from a retrospective cohort and animal model
Source: PLoS One. 2026 Jan 8;21(1):e0333081. doi: 10.1371/journal.pone.0333081 (PMC12782434; doi:10.1371/journal.pone.0333081)
Supplement: S3 Table — (DOCX) [file pone.0333081.s004.docx]

| **S3 Table. Basic information on pregnant women** [*‾ x*±*s*, *M* (*P*_25_, *P*_75_), *n* (%)] | | | | | |
| --- | --- | --- | --- | --- | --- |
| Group | Underweight  (n = 45) | Normal weight  (n = 229) | Overweight  (n = 57) | Obesity  (n = 22) | *P* value |
| Age (years) | 29.80±3.36 | 30.74±3.42 | 31.84±3.34^*^ | 31.09±3.18 | 0.024 |
| BMI (kg/m^2^) | 17.36 (17.15,18.03)^****^ | 20.94  (19.65,21.88)^****^ | 25.39  (24.77,26.45)^****^ | 29.69  (28.76,30.93)^****^ | < 0.0001 |
| Menstrual days (days) | 6.09±1.04 | 6.07±1.29 | 5.53±1.42^**^ | 5.95±1.33 | 0.037 |
| Menstrual cycle (days) | 30 (28,32) | 30 (28, 32) | 30 (29, 35) | 30 (29, 35) | 0.279 |
| Previous miscarriage | 1 (0.5,2) | 2 (1,2) | 2 (1,2) | 1 (0,2) | 0.099 |
| Adverse pregnancies | 1 (0.5,2) | 1 (1,2) | 1 (1,2) | 1 (0,2) | 0.132 |
| Assisted reproduction | 10 (22.2%) | 43 (18.8%) | 9 (15.8%) | 6 (27.3%) | 0.651 |
