# Supplementary figures and images for: Effects of maternal BMI on early pregnancy endocrine–metabolic function and offspring development: Evidence from a retrospective cohort and animal model
Source: PLoS One. 2026 Jan 8;21(1):e0333081. doi: 10.1371/journal.pone.0333081 (PMC12782434; doi:10.1371/journal.pone.0333081)

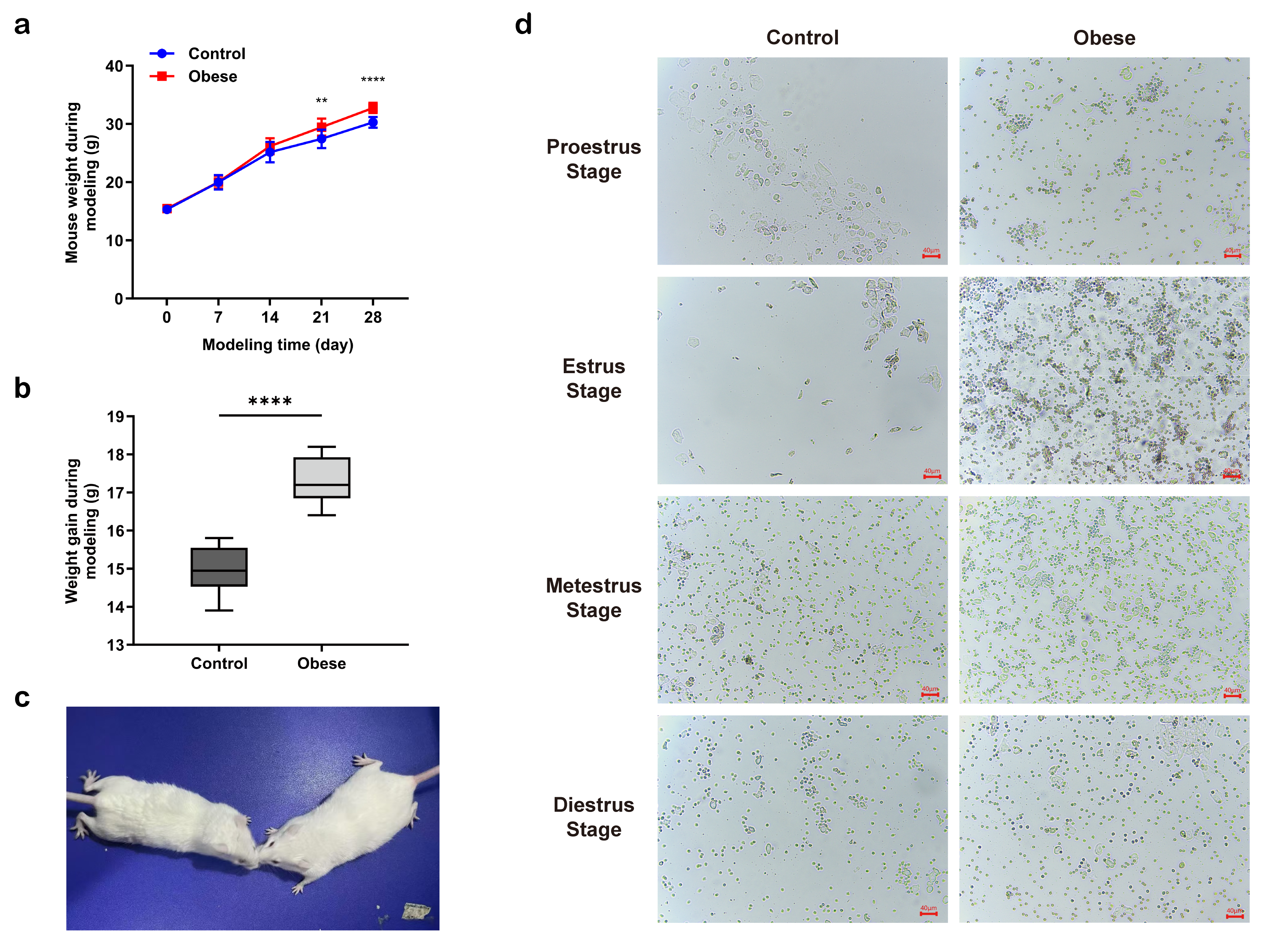

Supplement: S1 Fig — (a) Line graph showing body weight gain of female mice during the modeling period. **P < 0.01, ****P < 0.0001. (b) Box plot illustrating body weight increase in the two groups after modeling. ****P < 0.0001. (c) Morphological appearance of mice after modeling (left: control group; right: obese group). (d) Vaginal cytology smears obtained during the modeling process. Scale bar = 40 μm. (TIF) [file pone.0333081.s005.tif]
